# Educational App-Development needs to be informed by the Cognitive Neurosciences of Learning & Memory

**DOI:** 10.1038/s41539-018-0039-4

**Published:** 2018-12-21

**Authors:** T. P. Reber, N. Rothen

**Affiliations:** 1 Faculty of Psychology, Swiss Distance Learning University, Brig, Switzerland; 2 Department of Epileptology, University of Bonn, Bonn, Germany; 3 Department of Psychology, University of Bern, Bern, Switzerland

Be it intentionally or not: computers, tablets, and smartphones have become ubiquitous in schools and have been transforming educational practices at all ages and levels and almost all over the world.^1^ Whether this transformation is for the better or the worse, identifying the factors that distinguish between these two trajectories has been elucidated to a great extent already by many educational studies. By contrast, this topic is only beginning to attract attention from the neurocognitive research of learning and memory. In this piece, we argue that research into the effectiveness and side-effects of the use of information and communication technology (ICT) in education could profit greatly from established and highly relevant knowledge from the cognitive neurosciences of learning & memory. Solid, large-scale and especially long-term and experimental research into the application of this knowledge using ICT for education and ultimately an adoption of the resulting knowledge in ICT development and educational practice is urgently needed.^2^

While the industries and media are predominantly concerned with the fourth industrial revolution - the fusion of digital, biological, and physical technology - the educational sector seems to still be trying to keep up with the third, i.e., the digital revolution. Here, digitization refers to the process of transforming information stored in an analog format, e.g., letters printed in a textbook, into a digital format, i.e., bits and bytes stored on a modern computing device. The term digitalization, on the other hand, is used to refer to a broader range of phenomena related to societal changes of using modern computing devices as means to learn and interact with others. We collectively refer to end-user devices and networking infrastructure of modern computers (e.g., tablet computers and WIFI Networks) as information and communication technology (ICT).

Many agree that ICT offers great potential to assist learning in principle.^3^ Empirical evidence that convincingly demonstrates how ICT could live up to that potential, however, has been very scarce. Such demonstrations seem to be lacking because most of the empirical research focuses on the perception of and attitudes towards ICT by students and teachers. While we think that research into studying perceptions and attitudes towards ICT is important and worthwhile, we also think that it is necessary to complement this knowledge with studies that actually measure the effects of ICT-assisted learning on primary learning outcomes such as school-grades or other measures of skill and knowledge.^2^ Furthermore, it is vital to consider both positive and negative effects of ICT on learning outcomes. For example, playing computer-games designed to train executive functions in school-children had positive effects on school grades.^4^ Other studies, however, even provided evidence for detrimental effects of ICT use on learning & memory, general health, and wellbeing. For instance, a recent study finds that taking notes by hand is superior than using laptops.^5^ Also, rather than fostering learning, communication via online messaging among peers is perceived as a hindrance for getting homework done in college students^6^ and may even result in bad sleep quality when ICT use is high in school-aged children.^7^

In the few cases where studies were explicitly focusing on primary learning outcomes, a medium effect of using ICT for learning has been documented by a recent meta-analysis by Sung et al.^8^ Interestingly however, this effect dissipates and eventually vanishes in the even fewer studies assessing intervals longer than a year (for details see e.g., the meta-analysis by Bano and colleagues^2^). The initial boost in learning thus seems not due to exclusive features of ICT often believed to be beneficial for learning or the specific design features of the learning app in question. Rather it seems that initial excitement and boost in motivation of the students as they first got to use ICT in an educational setting is responsible for these positive effects. Considering the vast amounts of money spent on ICT in educational settings, relative to the cost of more traditional learning approaches (e.g., textbooks), this outcome is rather disappointingly sobering. Other meta-analyses paint similar pictures and add that explicit connections between pedagogical theories guiding app-development and specific features of the apps are mostly lacking.^8,9^ Ultimately, it is actually unclear how much is gained by expensive ICT-approaches over cheaper traditional learning approaches that follow well established principles of learning and memory.

The cognitive neuroscience of learning & memory has established knowledge on how to optimize learning in traditional learning settings.^10^ Here we briefly outline the four most prominent principles to adhere for optimizing the learning outcome. One of the most efficient ways to boost learning is to focus on the practice to actively retrieve (i.e., retrieval practice) rather than repeatedly, passively expose one-self to to-be-learned information. This so-called testing effect is not only operational within a given pool of learning items (“backward testing effect”) but more recently has also been shown to generalize to the acquisition of entirely new material (“forward testing effect”), suggesting a mechanism of “learning to learn”.^11^ For educational app-developers, this entails to engage app-users more frequently in testing rather than encoding situations. However, further research is needed to find the appropriate proportion of (passive) learning vs. testing phases. A second principle holds that learning works best when it is distributed over time, rather than crammed all up into one long session. Some of the specifics of how to optimally space learning episodes over time, especially also at longer retention intervals, have already been identified but the topic still remains an area of active research.^12^ Here, app-design could envisage appropriately scheduled reminders to learn and use the app. A third principle is to present learning material on multiple senses/channels simultaneously. This multimodal learning can be achieved by e.g. presenting a word written on the screen while the spoken representation of the word is delivered through the speakers of the device. Multimodal learning has been shown to recruit more widely distributed areas in brain than unimodal learning^13^ and may thus facilitate learning by providing many routes for storage and retrieval of to-be-learned information. Finally, learning & memory research has demonstrated that learning through feedback, especially when corrective, significantly boosts learning-efficiency.^14^ Thus, rather than penalize for errors, educators and developers of educational apps should encourage students to take risks and commit errors during learning as this benefits retention and decision making under high-stakes situations, as long as corrective feedback is provided.

Although all these principles are well established in the laboratory, they are vastly underexplored in real-life educational settings.^13^ Moreover, almost all studies on these principles were restricted to relatively short time intervals (less than half a year^2^). While these principles have been known by memory researchers for quite some time now, developers of ICT Learning apps seldomly refer to these principles when designing their apps. This apparent lack of transfer between basic memory researchers and app-developers may explain the lack of evidence for beneficial effects of classroom ICT above and beyond more traditional learning approaches. This lack of positive evidence should also be of interest for policymakers as total ICT exposure of students during teaching time is increasing and the related public costs such as to equip classrooms with ICT infrastructure are immense. Costs for ITC are estimated at 2.2 billion in 2011,^10^ rising up to 14 billion for K-12 and 12.8 Billion in 2018 for higher education in the US alone.^15^

To summarize, ICT use in education is growing almost exponentially and ICT is very expensive relative to analog learning means. A core problem is that memory-based learning such as e.g. vocabulary learning with ICT does not seem to be superior compared to more traditional approaches. A very likely reason for the failure of ICT to facilitate learning is that design of ICT applications is not informed by established scientific knowledge from basic cognitive neuroscience of memory. This research on learning and memory has identified highly efficient learning principles mostly in the laboratory. These principles, however, are vastly underexplored in real-world educational settings. Valuable efforts have been made but we are missing long-term studies with larger sample sizes to assess the potential real-life benefits of the learning principles identified in the laboratory.

There is a straightforward solution to these problems. Educational ICT focusing on memory-related learning such as e.g. foreign-language learning apps need to be developed on the basis of fundamental principles derived from cognitive neuroscience of memory. Theoretically motivated design features such as the specifics of the spacing of the learning episodes need to be varied within ICT application. This within-app variation of factors hypothesized to aid learning, and their interactions, can then be assessed in isolation and respective effect-sizes can be meaningfully compared. At the same time, these tools can be used to collect data in real-world educational scenarios, not only in the classroom setting but also under homework conditions. This will allow for the collection of large samples in long-term real-world scenarios. The richness of the resulting data bears the potential to answer many unresolved questions in the field of learning and memory.
